# The PIN domain endonuclease Utp24 cleaves pre-ribosomal RNA at two coupled sites in yeast and humans

**DOI:** 10.1093/nar/gkw213

**Published:** 2016-03-31

**Authors:** Graeme R. Wells, Franziska Weichmann, David Colvin, Katherine E. Sloan, Grzegorz Kudla, David Tollervey, Nicholas J. Watkins, Claudia Schneider

**Affiliations:** 1 Institute for Cell and Molecular Biosciences, Newcastle University, Newcastle upon Tyne NE2 4HH, UK; 2 Wellcome Trust Centre for Cell Biology, University of Edinburgh, Edinburgh EH9 3JR, UK; 3 MRC Institute of Genetics and Molecular Medicine, University of Edinburgh, Edinburgh EH4 2XU, UK

## Abstract

During ribosomal RNA (rRNA) maturation, cleavages at defined sites separate the mature rRNAs from spacer regions, but the identities of several enzymes required for 18S rRNA release remain unknown. PilT N-terminus (PIN) domain proteins are frequently endonucleases and the PIN domain protein Utp24 is essential for early cleavages at three pre-rRNA sites in yeast (A0, A1 and A2) and humans (A0, 1 and 2a). In yeast, A1 is cleaved prior to A2 and both cleavages require base-pairing by the U3 snoRNA to the central pseudoknot elements of the 18S rRNA. We found that yeast Utp24 UV-crosslinked *in vivo* to U3 and the pseudoknot, placing Utp24 close to cleavage at site A1. Yeast and human Utp24 proteins exhibited *in vitro* endonuclease activity on an RNA substrate containing yeast site A2. Moreover, an intact PIN domain in human UTP24 was required for accurate cleavages at sites 1 and 2a *in vivo*, whereas mutation of another potential site 2a endonuclease, RCL1, did not affect 18S production. We propose that Utp24 cleaves sites A1/1 and A2/2a in yeast and human cells.

## INTRODUCTION

**Figure 1.**
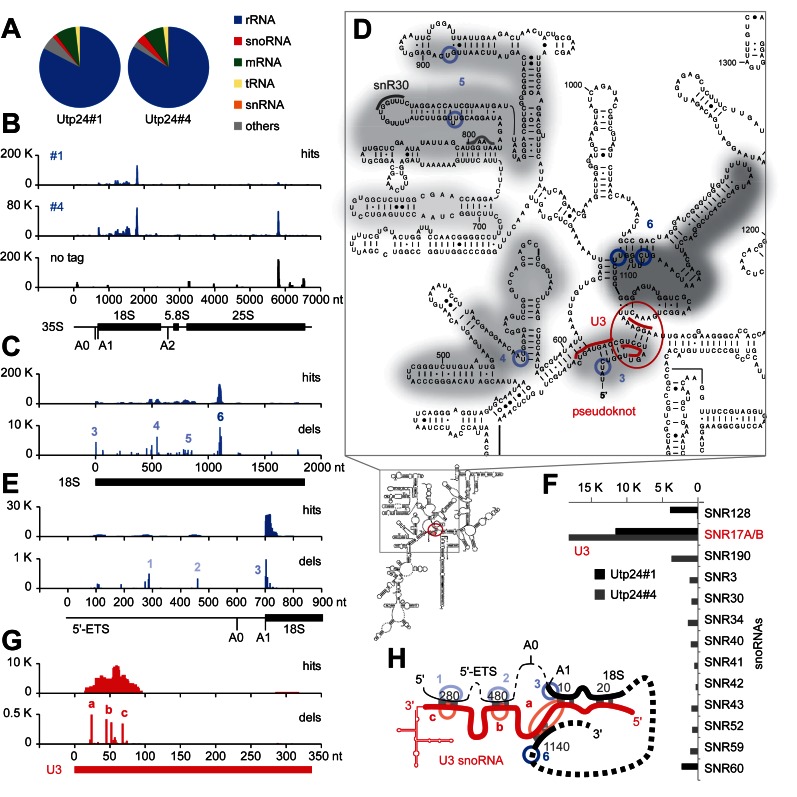
RNA crosslinking sites of yeast Utp24 on (pre-) rRNA and the U3 snoRNA. Illumina sequencing of cDNA libraries generated from crosslinked and subsequently trimmed RNAs recovered with purified Utp24 protein. Normalized data are plotted as reads per million mapped sequences. (**A**) Transcriptome-wide binding profiles. Data of two replicate experiments were mapped to the yeast genome using Novoalign. A total of 979 330 mapped reads were recovered for data set Utp24#1 and 1383819 for data set Utp24#4, respectively. Pie charts illustrate the proportion of all sequences mapped to functional RNA classes (indicated on the right). (**B**) Sequences from data sets Utp24#1 and Utp24#4 and a control experiment with a non-tagged strain (no tag) were aligned with the rDNA (RDN37-1) encoding the 35S pre-rRNA. The frequency of recovery is plotted as hits (total reads) for each individual nucleotide. (**C**, **E** and **G**) RNA binding profiles on (C) the 18S rRNA, (E) the 5′-external transcribed spacer (5′-ETS) and (G) the U3 snoRNA. Hits (upper panels): total reads; dels (lower panels): mutations and microdeletions representing precise binding sites. Microdeletion peaks in the (pre-) rRNA (1-6, blue) and the U3 snoRNA (a–c, red) are labeled. The position of the mature 18S, 5.8S and 25S rRNAs (B, C and E) or the U3 snoRNA (G) are indicated by thick lines. (**D**) Predicted secondary structure of the mature 18S rRNA in *S. cerevisiae*. Utp24 crosslinking sites are marked on the sequence and shades indicate peak height with the highest peak shown in dark grey. Microdeletion peaks (see panel C) are highlighted by shaded blue circles. Binding sites for snoRNAs U3 around the central pseudoknot (red) and snR30 in the ES6 region (grey) are also indicated. (**F**) The distribution of hits mapping to crosslinked snoRNAs is plotted. (**H**) Schematic representation of base-pairing interactions between the pre-rRNA (black) and the U3 snoRNA (red) during pseudoknot formation. The approximate positions of Utp24 microdeletion peaks are indicated in blue (1–6, pre-rRNA, see panels C and E) or red (a–c, U3 snoRNA, see panel G).

The eukaryotic rRNAs are processed from the 35S (*Saccharomyces cerevisiae*) or 47S (*Homo sapiens*) rRNA precursors (pre-rRNAs) by endonucleolytic cleavages and exonucleolytic trimming, with concomitant removal of external (5′-ETS, 3′-ETS) and internal (ITS1, ITS2) transcribed spacer sequences (Figure 1 and Supplementary Figure S1) (1).

Early pre-rRNA cleavages, at sites called A0, A1 and A2 in yeast and A0, A1/1 and 2a/E in humans, are important for 18S rRNA maturation. These events require a large ribonucleoprotein complex, the small subunit (SSU) processome (or 90S pre-ribosome) (2). A key component of the SSU processome is the U3 small nucleolar (sno)RNA, which base-pairs with the 5′-ETS and 18S rRNA elements to chaperone the formation of the conserved pseudoknot, a key structural feature of the 40S ribosomal subunit (2,3).

Loss of many different SSU processome components blocks pre-rRNA cleavage at sites A0, A1/1 and A2/2a, making it unclear which factor is the active nuclease. However, two evolutionarily conserved factors feature protein domains linked to RNA processing. Yeast Utp24/Fcf1 and human UTP24 harbor PIN (PilT N-terminus) endonuclease domains (4), whereas yeast Rcl1 and human RCL1 exhibit RNA cyclase-like protein folds (5). A PIN domain is also found in the endonuclease Nob1, which catalyzes the final cytoplasmic maturation step at the 3′-end of the 18S rRNA (site D) in yeast (6–8).

Yeast Utp24 and Rcl1 are essential for growth and conditional depletion of either protein inhibits A0, A1 and A2 cleavage (4,5,9). However, mutation of the PIN domain of Utp24 specifically inhibited cleavage at sites A1 and A2, while cleavage at site A0 was unaffected (4), and the mutant was dominant negative when expressed together with intact Utp24. These observations suggested that the presence of Utp24 is required for SSU processome function in A0-A2 cleavage, whereas the PIN domain harbors the catalytic activity for A1 and A2 cleavage (4), but nuclease activity was not demonstrated. Mutation of Rcl1 also inhibited site A2 cleavage, with less effect at A0 and A1 (5,9,10), and recombinant Rcl1 was reported to cleave pre-rRNA transcripts containing yeast site A2, consistent with direct endonuclease activity (9).

In mammals, the equivalent pre-rRNA cleavages also require the presence of UTP24 (FCF1 in mouse) and RCL1 (11–14) and the putative catalytic activity of human UTP24 was very recently linked to site 1 cleavage (15). However, the catalytic roles of UTP24 and RCL1 with respect to 2a cleavage have not been established.

In yeast, mutational and kinetic analyses indicate that processing at sites A1 and A2 is tightly coupled and mainly co-transcriptional (16,17). In vertebrates, pre-rRNA processing appears to be largely post-transcriptional and A′ processing near the 5′-end of the 5′-ETS in humans, a cleavage site not present in yeast, appears to be the only co-transcriptional event (18). Cleavage at site 2a within the human ITS1 (equivalent to yeast A2) is part of a minor pathway in humans (Supplementary Figure S1) (11,12,19). However, no precursors processed at site 2a but still containing 5′-ETS sequences are detected, indicating that A0, 1 and 2a cleavages are coupled within the SSU processome, as in yeast.

Here, we present a combination of *in vivo* and *in vitro* approaches to clarify the roles of Utp24 and Rcl1 in yeast and human ribosome biogenesis. *In vivo* RNA–protein crosslinking studies (CRAC) generated a transcriptome-wide RNA binding profile for yeast Utp24, which provides fresh insights into its function. We also performed *in vitro* assays using recombinant wild-type (WT) and mutant Utp24 and Rcl1 proteins to assess their cleavage activity on pre-ribosomal RNA. Finally, we established RNAi-rescue systems in HEK293 cells to study the effect of presumably catalytically inactive UTP24 and RCL1 mutants on pre-rRNA cleavage in the human system.

## MATERIALS AND METHODS

### Yeast strains and methods

*S. cerevisiae* strains (Supplementary Table S1) were constructed by standard methods (20). Cultures were grown at 30°C in medium containing 0.67% nitrogen base (Difco) and 2% glucose or 2% galactose. Strains for crosslinking studies expressed genomically encoded, C-terminal HTP-tagged proteins under the control of their endogenous promoter.

### CRAC and data analysis

The CRAC method was performed as previously described (21,22), see Supplementary Figure S2A. To generate RNA–protein crosslinks, actively growing yeast cultures in SD medium (OD_600_ ∼0.5) were UV-irradiated in a 1.2 m metal tube for 100 s at 254 nm (22). Illumina sequencing data were aligned to the yeast genome using Novoalign (http://www.novocraft.com). Bioinformatics analyses were performed as described (23). The Illumina sequencing data from this publication have been submitted to the GEO database (http://www.ncbi.nlm.nih.gov/geo/) and assigned the identifier GSE75991.

### Cloning and mutagenesis

The open reading frames for Rcl1, Bms1, Utp24 or UTP24 were amplified from yeast genomic DNA or human cDNA adding restriction sites (Supplementary Table S2) and cloned into pET100 vectors (Invitrogen). The constructs were used for *in vitro* translation in the presence of [^35^S] methionine (TNT, Promega) or subcloned into pGEX-6P1 vectors to express and purify Glutathione *S* - transferase (GST) - tagged recombinant proteins from *E. coli* using standard techniques. The C-terminally HTP-tagged Rcl1 gene was amplified by PCR from yeast genomic DNA (Strain Rcl1-HTP) adding restriction sites to the 5′-end of the gene and the 3′-end of the HTP-tag, respectively, and cloned into pRS316. The coding sequences of UTP24 and RCL1 were altered to make them resistant to the siRNAs (Supplementary Table S3) used to deplete the endogenous mRNAs. These constructs (IDT) were amplified by PCR and cloned into the pcDNA5/FRT/TO vector (Invitrogen) containing 2x N-terminal FLAG tags under the control of a tetracycline-inducible promoter. Point mutations were generated by QuikChange site-directed mutagenesis using overlapping primers (Supplementary Table S2) and verified by sequencing.

### Cell culture and RNAi

Constructs were transfected into Flp-In T-Rex HEK293 cells. Stably transfected cells were selected as described by the manufacturor (Invitrogen) and cultured according to standard protocols. Expression of exogenous proteins was induced by addition of tetracycline (UTP24; 1 mg/ml, RCL1; 0.01–0.1 mg/ml). Cells were transfected with siRNA duplexes (Supplementary Table S3) using Lipofectamine RNAiMAX transfection reagent (Invitrogen) and harvested after 72 h depletion.

### RNA analysis

RNA was extracted from HEK293 cell pellets using TRI reagent (Sigma-Aldrich). For northern blot analysis, 2 μg of total RNA was separated on 1.2% glyoxal-agarose gels, transferred to nylon membrane and hybridized with 5′-end labeled oligonucleotide probes (Supplementary Table S2). For primer extension analysis, 1 μg of total RNA was converted into cDNA using Superscript III (Invitrogen) and 5′-end labeled oligonucleotide probes and separated on 10% PAA/8 M urea sequencing gels. Results were visualized using a PhosphorImager (Typhoon FLA9000; GE Healthcare). ImageQuant (GE Healthcare) was used to quantify northern blot data, which were normalized to levels of the 47S/45S pre-rRNAs.

### *In vitro* RNA cleavage assay

Recombinant proteins were expressed and purified as described in (24), including 1 mM MnCl_2_ in all media and buffers. Nuclease assays were performed in 10 mM Tris/HCl pH 7.6, 100 mM NaCl, 2 mM dithiothreitol, 100 μg ml^−1^ bovine serum albumin (BSA), 0.8 unit μl^−1^ RNasin, 4.5% glycerol, 0.05% Tween20, 10 ng μl^−1^*E. coli* tRNA and 5 mM MnCl_2_. A total of 10 μl reactions containing ∼20 pmol of protein were pre-incubated for 5 min at 30°C. The only exception was for the experiment shown in Supplementary Figure S4, for which detailed conditions are described in the supplementary figure legend S4. *In vitro* transcribed pre-rRNA substrates (0.125 pmol) containing yeast sites D and A2 (35S + 2301 − 2844) or yeast sites A0 and A1 (35S + 335 − 1146) was added and incubated for 1 h at 30°C. Following proteinase K digestion (30 min at 37°C), the RNA was extracted, precipitated and analyzed by primer extension using probes yD-RT, yA2-RT or yA1-RT, respectively. Products were resolved on 10% PAA/8 M urea sequencing gels and visualized by autoradiography.

### Protein–protein interaction studies and purification of Rcl1-containing complexes

GST-bait proteins (∼50 pmol) were immobilized on glutathione sepharose and incubated with [^35^S] *in vitro* translates for 1 h at 4°C in Buffer NB (20 mM Tris/HCl pH 7.6, 150 mM NaCl, 8.7% glycerol and 0.1% Tween20). The beads were washed five times with buffer NB. Retained proteins were separated by SDS-PAGE and visualized by autoradiography. Affinity-purification of Rcl1-HTP complexes (from 500 ml of yeast culture each) was performed essentially as described in (25). Complexes were purified on IgG sepharose at 150 mM NaCl and eluted by TEV protease cleavage. Co-purifying proteins and RNAs were analyzed by immunoblotting or northern blotting, respectively (Supplementary Tables S4 and S2).

### Yeast immunofluorescence

Yeast strains expressing HA-tagged Rcl1 under control of the repressible *GAL10* promoter were transformed with plasmids encoding His_6_-TEV-protein A (HTP) tagged forms of Rcl1 and Rcl1_RDK_ and grown in minimal medium containing glucose for 6 h to deplete the endogenous protein. Cells at mid-log phase were harvested and fixed in 4% formaldehyde in phosphate buffered saline (PBS) (15 min at RT), washed with PBS and then incubated for 45 min at 30°C in buffer B (0.1 M potassium phosphate pH 7.5, 1 M sorbitol, 10 mM DTT) containing 50 U/ml Zymolase. Cells were washed and incubated in batch with the antibodies diluted in PBS + 5% milk (Supplementary Table S4), washed with PBS containing DAPI (4′,6′-diamidino-2-phenylindole) and mounted onto poly-lysine coated coverslips using Vectorshield. All images were obtained using a Zeiss Axiovert 200 microscope with Plan-Apochromat x100 1.4NA objective, Axiovision software and an Axiocam monochrome camera, and processed in Photoshop (Adobe).

## RESULTS

### Yeast Utp24 crosslinks in close vicinity to site A1, to the U3 snoRNA and pre-rRNA elements required for pseudoknot formation

To dissect the roles of the two yeast candidate pre-rRNA endonucleases Rcl1 and Utp24, we applied *in vivo* RNA–protein crosslinking (CRAC) to identify their RNA binding sites (21) (Figure 1 and Supplementary Figure S2). C-terminal HTP-tagged (His_6_–TEV–protA) Rcl1 and Utp24 were expressed from the chromosomal locus under control of the endogenous promoter. Rcl1-HTP supported WT growth, while the Utp24-HTP strain exhibited a mild growth defect. Actively growing cells were UV-irradiated as described (22) and RNA fragments crosslinked to the purified proteins were isolated and analyzed as outlined in Supplementary Figure S2A. Protein recovery was verified by western analysis (Supplementary Figure S2B). Rcl1 purified well, but crosslinked poorly, and no pre-rRNA target sequence was significantly enriched in every experiment. We were, however, able to reproducibly identify Utp24 RNA crosslinking sites in four independent CRAC experiments. Results from two representative data sets are presented in Figure 1 and Supplementary Figure S2.

Transcriptome-wide RNA binding profiles of Utp24 are shown in Figure 1A. The majority of reads (>83%) identified in both Utp24 data sets were mapped to the (pre-)rRNA, with lower numbers of hits in snoRNAs. Hits were also recovered in mRNAs, but no individual mRNA emerged as a likely Utp24 target. Consistent with its known role in SSU biogenesis, Utp24 was predominately associated with sequences within the 18S rRNA (Figure 1B and C). Importantly, this crosslinking profile was not seen in a CRAC experiment performed with an untagged control strain (Figure 1B). The peak near the 3′-end of the 25S rRNA represents a common CRAC contaminant (21).

In CRAC, microdeletions and/or mutations are often introduced at the site of crosslinking during cDNA preparation and can be used to map precise protein-binding sites (26). Sites of microdeletion (Figure 1C and D) were seen at 18S +3, adjacent to cleavage site A1 (peak 3, see Supplementary Figure S2C for higher resolution), around 18S + 545 (peak 4) and around 18S + 820 and 18S + 895 within expansion segment 6 (ES6) (peak 5), close to the binding site for the snoRNA snR30, which is also required for cleavage at sites A0-A2 (27). The highest peak was located at 18S + 1103 in the vicinity of the 3′-side of the central pseudoknot (peak 6). Sites of microdeletion were also identified in the flanking 5′-ETS (Figure 1E) around 35S + 288 (peak 1) and 35S + 460 (peak 2). Strikingly, these positions in the 5′-ETS correspond to the base-pairing sites for the U3 snoRNA 3′-hinge and 5′-hinge regions, respectively (3).

Analysis of snoRNAs crosslinked to Utp24 (Figure 1F) revealed specific enrichment for the U3 snoRNA (encoded by the genes *SNR17A/B*). Utp24 predominately crosslinked to a U3 region (U3 +15−90), which undergoes multiple interactions with the pre-rRNA. Significant peaks of microdeletions in U3 (Figure 1G and H) were seen at +45 and +52 (peaks b, 5′-hinge) and +68 (peak c, 3′-hinge) that base-pair to 5′-ETS regions at +480 and +280, respectively (2,3). The highest peak of microdeletions (peak a) was at +24, a region of U3 predicted to base-pair with 18S +1140 on the 3′-side of the pseudoknot.

We conclude that Utp24 binds both to the U3 snoRNA and to the corresponding U3-binding sequences in the mature 18S rRNA and the 5′-ETS, interactions that are essential for pseudoknot formation and coupled cleavage of sites A0, A1 and A2. Moreover, the binding of Utp24 at 18S +3 is consistent with a direct role in cleavage at site A1, since the precise location of cleavage is partly defined with respect to this position (28).

### Utp24 cleaves site A2 in a yeast pre-rRNA substrate *in vitro*

**Figure 2.**
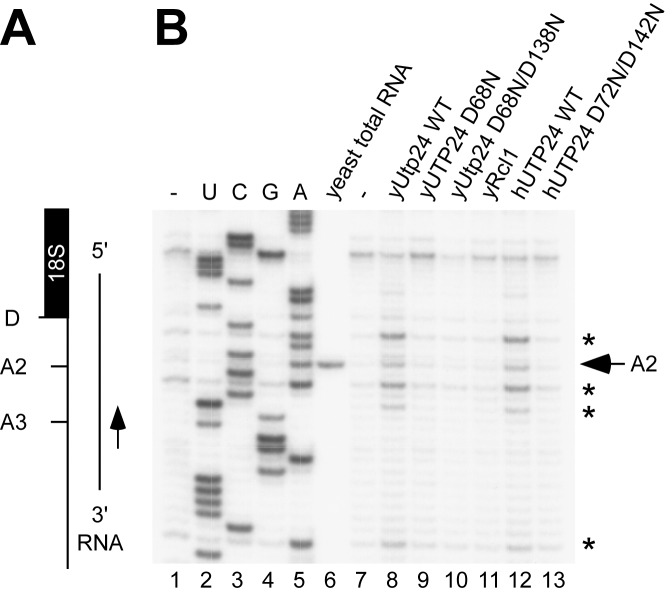
Yeast and human recombinant Utp24 proteins cleave at yeast site A2 *in vitro*. (**A**) Schematic of the RNA substrate mimicking a pre-rRNA fragment before A2 cleavage (yeast 35S +2301-2844). (**B**) *In vitro* transcribed RNA was incubated without recombinant protein (-), wild-type (WT) or mutant Utp24, WT Rcl1 or WT or mutant UTP24 in the presence of 5 mM Mn^2+^ and analyzed by primer extension. The position of the primer is shown in panel A. Non-treated RNA substrate was used to generate a sequencing ladder and endogenous cleavage at site A2 in yeast total RNA is indicated. Recombinant Utp24-mediated cleavages at site A2 and 5′C/A3′ sequences are marked by an arrow and asterisks, respectively.

The predicted catalytic center within the Utp24 PIN domain is characterized by four acidic residues (DEDD), which are conserved in all kingdoms of life (29). We therefore assessed the *in vitro* activities of yeast Utp24 and human UTP24 in cleavage assays (Figure 2, Supplementary Figures S3 and S4). WT and PIN domain mutant proteins were expressed in *E. coli* with N-terminal tags containing GST. Double (D72N/D142N) catalytic site mutations in UTP24 were based on yeast Utp24 mutations (D68N, D138N) that cause A1 and A2 cleavage defects *in vivo* (4). The N-terminal GST tag was removed by prescission protease (PP) cleavage following purification (Supplementary Figure S3A).

The proteins were tested in nuclease assays on *in vitro* transcribed RNA containing yeast site A2 (Figure 2A) or A1 (Supplementary Figure S3B). Assays were performed in the presence of 5 mM Mn^2+^, which is required for the *in vitro* activity of other PIN domain nucleases (7,25,30). The RNA was analyzed by primer extension using a labeled oligonucleotide downstream of the cleavage site.

WT yeast and human Utp24 both cleaved the RNA substrate containing site A2, giving very similar products (Figure 2B, lanes 8 and 12). In contrast, no cleavage activity was observed for the predicted catalytically inactive Utp24 PIN mutants (Figure 2B, lanes 9, 10 and 13), while a different PIN domain endonuclease, Nob1, cleaved site D but not A2 (Supplementary Figure S3D). Utp24-mediated cleavage did not occur exclusively at site A2, but also at several other places with the same sequence context (5′C*/*A3′, where '/' equals the site of cleavage) around the *bona fide* cleavage site. In contrast, we did not detect clear cleavage at site A1 (Supplementary Figure S3B, see Discussion). An evolutionarily conserved ACAC motif at site A2 was previously identified as a specificity feature for yeast A2 cleavage *in vivo* (31). Since the *in vitro* cleavage pattern was identical for yeast and human Utp24 in our assays, this appears to be a conserved feature of Utp24 PIN nuclease activity.

Yeast Rcl1 was also expressed and purified with a cleavable GST-PP tag (Figure 2 and Supplementary Figure S3A), but no *in vitro* cleavage activity was detected in the presence of 5 mM Mn^2+^ (Figure 2B, lane 11). In an effort to reproduce the reported nuclease activity of Rcl1 (9), we dialyzed the yeast Rcl1 protein, as well as WT and mutant Utp24 proteins, into the buffer optimized for Rcl1 activity (K. Karbstein, personal communication) and performed the nuclease assay in the presence of 10 mM Mg^2+^. Under these conditions, apparent cleavage at site A2 was observed (Supplementary Figure S4), as reported. However, cleavage was observed with each of the recombinant proteins, including the catalytically inactive Utp24 D68N mutant, indicating that cleavage associated with addition of Utp24 was not specific. We were unable to express and purify a mutant of Rcl1 that is predicted to lack catalytic activity, and were therefore unable to determine whether this is also the case for Rcl1.

### An intact PIN domain in human UTP24 is required for accurate cleavages at sites 1 and 2a, but not A0, *in vivo*

**Figure 3.**
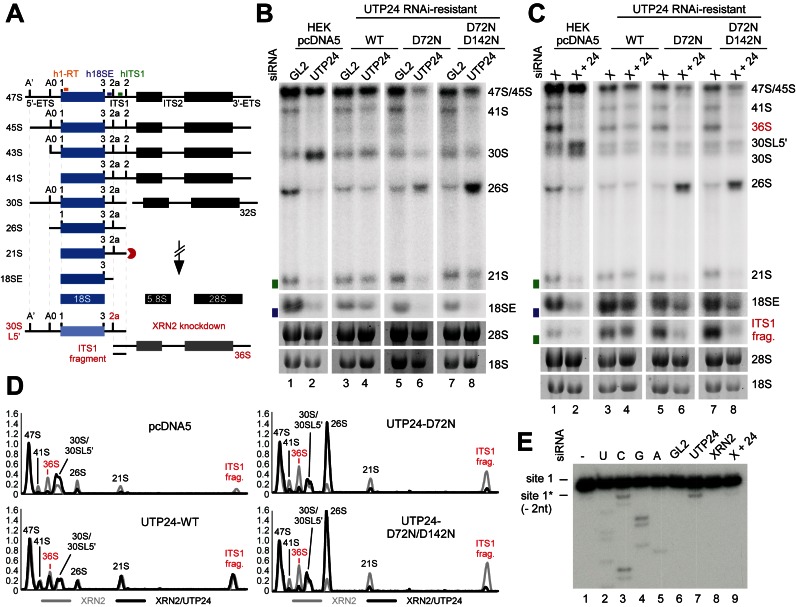
An intact UTP24 PIN domain is required for efficient site 1 and 2a cleavages in the human pre-ribosomal RNA. (**A**) Key steps in human ribosome biogenesis. RNA intermediates of the minor, 2a-dependent pathway accumulating in the absence of XRN2 are shown in red. Radiolabeled probes used for primer extension (h1-RT; orange) or northern blotting (h18SE; purple and hITS1; green) are marked above the 47S precursor. ETS: external transcribed spacer; ITS: internal transcribed spacer. (**B**) HEK293 cells were stably transfected with plasmids expressing the FLAG tag (pcDNA5) or WT or mutant forms of FLAG-UTP24 (D72N, D72N/D142N). RNA from control cells (GL2) or those depleted of endogenous UTP24 (UTP24) by RNAi, was analyzed by northern blotting using probes hybridizing to the 5′-end of ITS1 (h18SE, purple rectangle) or downstream of 2a (hITS1, green rectangle). Pre-rRNAs were detected using a PhosphorImager and total rRNA (28S/18S) was visualized by ethidium bromide staining. RNA species are marked on the right. (**C**) RNA extracted from HEK293 cell lines as in panel B, but depleted of XRN2 alone (XRN2) or XRN2 and UTP24 (X+24) were analyzed as in B. (**D**) RNA levels from panel C were normalized to the 47S/45S pre-rRNAs and plotted for each XRN2-UTP24 double knockdown (black) and the single XRN2 knockdown (grey). The identity of each peak is indicated. Red: RNA species accumulating in the absence of XRN2. (**E**) RNA extracted from HEK293 cell expressing the FLAG-UTP24 D72N mutant, either treated with control siRNA (GL2) or depleted of UTP24 (UTP24), XRN2 (XRN2) or both (X+24), was analyzed by primer extension using probe h1-RT (panel A). Total RNA from control cells (GL2) expressing the FLAG tag alone (pcDNA5) was used to generate a sequencing ladder. Positions of the natural site 1 and 2 nt downstream are indicated on the left.

Yeast cleavage sites A0–A2 have direct counterparts in humans called sites A0, 1 and 2a (Supplementary Figure S1). RNAi-mediated knockdown of human UTP24 caused 30S pre-rRNA accumulation, indicating that UTP24 is required for these cleavages (11,13). To investigate the putative catalytic role of UTP24, we established an RNAi-rescue system in HEK293 cells. Cells stably expressing the FLAG-tag (pcDNA5) or FLAG-tagged forms of either WT UTP24, or UTP24 with single (D72N) or double (D72N/D142N) catalytic site mutations were generated (Figure 3 and Supplementary Figure S5). These carried silent mutations in the open reading frames rendering them resistant to RNAi-mediated depletion of the endogenous protein. The RNAi-resistant proteins were expressed at endogenous levels using a titratable *TET* promoter. Cells were transfected with either a siRNA specifically targeting endogenous UTP24 or a control siRNA targeting firefly luciferase (GL2) (32). After siRNA treatment for 72 h, the expression of endogenous and FLAG-tagged UTP24 proteins was analyzed by immunoblotting (Supplementary Figure S5A). The UTP24-specific siRNA significantly reduced endogenous protein levels, whereas the RNAi-resistant tagged UTP24 proteins were unaffected.

Total RNA was extracted from RNAi-treated cells and pre-rRNA processing analyzed by northern hybridization using probes complementary to the 5′-end of ITS1 (‘h18SE’) or downstream of the 2a cleavage site (‘hITS1’) (Figure 3B and Supplementary Figure S5B). Depletion of UTP24 resulted in 30S accumulation (Figure 3B, lane 2), and this phenotype was almost completely rescued by expression of the WT protein (lane 4). Complementation with UTP24 D72N or D72N/D142N mutants caused accumulation of the 26S pre-rRNA (lanes 6 and 8), indicative of strongly reduced cleavage at sites 1 and 2a. No dominant negative phenotypes were observed upon expression of the PIN mutants in the presence of the endogenous protein (lanes 5 and 7).

The 26S RNA accumulation indicated that the catalytic activity of human UTP24 is required for site 1 and 2a cleavage *in vivo*, while cleavage at site A0 is unaffected. However, 2a cleavage cannot readily be directly assessed because the 36S pre-rRNA and the excised ITS1 fragment, the only intermediates specific to the ‘minor’ pathway (lighter shades in Figure 3A), are barely detectable in control cells. However, depletion of the 5′–3′-exonuclease XRN2 stimulates processing through this pathway (11,19,33). We, therefore, performed UTP24 knockdown and complementation in combination with XRN2 depletion. Pre-rRNA intermediates were detected by northern hybridization with probe hITS1 (Figure 3C), and quantified using a PhosphorImager. Levels were normalized to the 47S/45S pre-rRNA and plotted to compare the single XRN2 and UTP24/XRN2 double knockdowns (Figure 3D). Depletion of XRN2 alone caused strong accumulation of the 36S pre-rRNA and the ITS1 fragment accompanied by the appearance of the 30SL5’ precursor (also referred to as 34S (12) (Figure 3C, lanes 1, 3, 5 and 7), reflecting an A’ cleavage defect. Double-knockdown of UTP24 and XRN2 (lane 2) resulted in a strong decrease in the 36S precursor and the ITS1 fragment compared to the single XRN2 knockdown, while expression of the WT UTP24 protein rescued the 2a processing defect (lane 4). Importantly, the 36S pre-rRNA and the excised ITS1 fragment were also severely reduced in double knockdown cells expressing UTP24 D72N (lane 6) or UTP24 D72N/D142N (lane 8), whereas 26S pre-rRNA levels were strongly increased.

Low levels of the 21S precursor were detected in UTP24 PIN mutant cells, suggesting that an alternative mechanism can generate the 18S rRNA 5′-end (site 1) in the absence of endonuclease cleavage. XRN2 degrades the 3′-fragment of the 5′-ETS (‘ETS3’) (13) and might also digest the 5′-ETS back to site 1 following A0 cleavage. To test this model, RNA was extracted from cells expressing UTP24 D72N, treated with a siRNA against endogenous UTP24, XRN2 or both, and analyzed by primer extension through site 1 (Figure 3E). The major stop at site 1 reflects mature 18S rRNA produced prior to the siRNA treatment. In cells treated with the UTP24 siRNA (lane 7), a weaker primer extension stop was visible two nucleotides downstream of site 1. This was significantly reduced in the UTP24/XRN2 double knockdown (lane 9) and absent when the catalytically inactive UTP24 was not expressed (Supplementary Figure S5C). We speculate that in the absence of site 1 cleavage, XRN2 degrades the 5′-ETS region, but can be blocked by catalytically inactive UTP24 bound at the 5′-end of 18S, leading to the observed truncated product.

Our analyses indicate that point mutations in the PIN domain of UTP24 block cleavage at sites 1 and 2a in human pre-rRNA, while cleavages at sites A0 and A’ are unaffected.

### Mutation of the proposed pre-rRNA substrate binding site within human RCL1 does not affect 18S production or 2a cleavage

**Figure 4.**
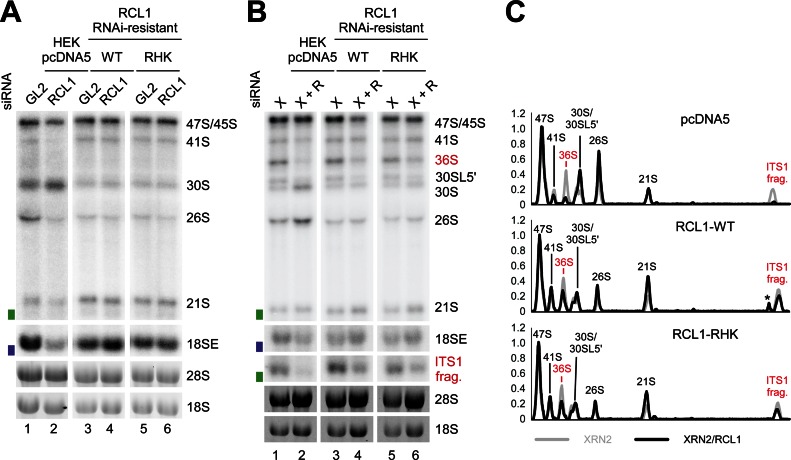
An intact RCL1 RHK domain is not required for human 18S production. (**A**) HEK293 cells were stably transfected with plasmids expressing the FLAG tag alone (pcDNA5) or WT FLAG-RCL1 or the FLAG-RCL1-RHK mutant (RHK). The FLAG-RCL1 mRNAs were rendered resistant to the RCL1 siRNA. RNA was extracted from control cells (GL2), or those depleted of endogenous RCL1 (RCL1) by RNAi and analyzed by northern blotting using a probe hybridizing to the 5′-end of ITS1 (h18SE, purple rectangle) or downstream of site 2a (hITS1, green rectangle). (**B**) RNA extracted from cells as listed in panel A, but depleted of XRN2 alone (XRN2) or XRN2 and RCL1 (X+R), was analyzed as in A. (**C**) The levels of the pre-rRNA intermediates from panel B were normalized to the 47S/45S precursors and plotted for each XRN2-RCL1 double knockdown (black) and the single XRN2 knockdown (grey). The identity of each peak is indicated. RNA intermediates accumulating in the absence of XRN2 are shown in red. Asterisk: non-specific signal.

Yeast Rcl1 was also reported to cleave at site A2 (9), suggesting the possibility of redundant activities. To determine whether RCL1 participates in human site 2a cleavage, we established an RCL1 RNAi-rescue system in HEK293 cells, as described for UTP24 (Figure 4 and Supplementary Figure S6). Endogenous and FLAG-tagged RCL1 levels were monitored by immunoblotting (Supplementary Figure S6A). RNAi-mediated depletion of RCL1 caused 30S accumulation (Figure 4A, lane 2), that was rescued by expression of RNAi-resistant FLAG-tagged WT RCL1 (Figure 4A, lane 4).

In yeast Rcl1 an ‘RDK-AAA’ mutant showed a strong A2 cleavage defect, which was proposed to be due to impaired pre-rRNA substrate binding (9). A stable cell line was constructed expressing RCL1 with the equivalent ‘RHK’ residues (R329, H330, K332) changed to alanines. Following RCL1 depletion, RCL1_RHK-AAA_ fully rescued the 30S phenotype, showing that integrity of the proposed RNA substrate-binding pocket is not required for human 18S maturation (Figure 4A, lane 6).

As noted above, XRN2 knockdown increases dependency on 2a cleavage, so the RCL1 knockdowns were repeated in combination with XRN2 depletion (Figure 4B). Following RNAi-mediated knockdown of XRN2 and RCL1, WT RCL1 and the RCL1_RHK_ mutant again rescued the pre-rRNA processing phenotype to a similar extent (Figure 4B, lanes 4 and 6). Quantification of pre-rRNA levels normalized to the 47S/45S pre-rRNAs confirmed that the 2a-dependent pre-rRNA intermediates, the 36S pre-RNA and the ITS1 fragment, are restored to similar levels in cells expressing WT and mutant RCL1, but severely reduced in cells expressing the FLAG-tag only (Figure 4C).

### Yeast Rcl1 mutations impair interactions with Bms1, integration into the SSU processome and subcellular localization

**Figure 5.**
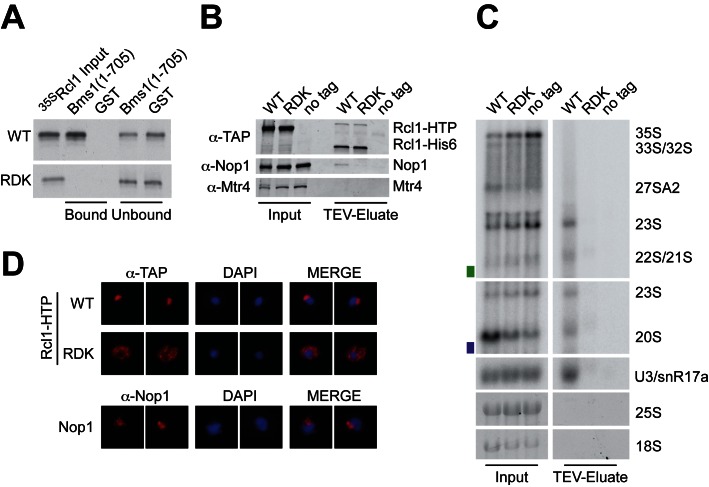
The yeast Rcl1_RDK_ mutant fails to interact with Bms1 *in vitro* and interferes with SSU processome integration and nucleolar localization *in vivo*. (**A**) Recombinant GST-tagged Bms1^(aa1-705)^ and GST were coupled to glutathione sepharose and incubated with WT and mutant Rcl1 generated by *in vitro* translation in the presence of [^35^S] methionine. Bound material was eluted under denaturing conditions, separated by SDS-PAGE and analyzed by autoradiography. A total of 10% of the input and unbound material is also loaded. (**B** and **C**) Rcl1-containing complexes were purified on IgG sepharose and eluted by TEV protease cleavage. (B) Co-purifying proteins were analyzed by SDS-PAGE and immunoblotting using antibodies against the C-terminus of the Rcl1-HTP tag after TEV cleavage (anti-TAP), the box C/D snoRNP component Nop1, or Mtr4 as a control. A total of 2.5% of the input material is also loaded. (C) Co-purified RNA was extracted and analyzed by northern blotting using a probe hybridizing to the U3 snoRNA or two regions within the pre-rRNA (yA2-A3, green rectangle or yD-A2, purple rectangle; see Supplementary Figure S1A for location of the probes). Mature 18S and 25S rRNAs were visualized by ethidium bromide staining. A total of 1% of the input material is also loaded. (**D**) Yeast strains expressing plasmid-encoded HTP-tagged WT Rcl1 (top row) or the Rcl1_RDK_ mutant (middle row) were harvested and processed for immunofluorescence miscroscopy using the anti-TAP antibody. Lower row: Cells stained with an antibody against endogenous Nop1 as a nucleolar marker. From left to right: Immunofluorescence signal (red), DAPI (blue) and the merged image.

The finding that the human equivalent of the yeast Rcl1_RDK_ mutant supports pre-rRNA processing prompted us to re-examine the ribosome synthesis defect in yeast (Figure 5). Recent mutational analysis of Rcl1 based on the crystal structure of the yeast Rcl1-Bms1 dimer showed that mutation of R237, within the RDK motif, disrupts interaction with Bms1 (10). The Rcl1-Bms1 interaction is required for nuclear import of Rcl1 (34) and, consistent with this, an R237A mutation severely impaired the nucleolar localization of yeast Rcl1 (10).

WT Rcl1 and Rcl1_RDK_ were generated by *in vitro* translation in the presence of [^35^S] methionine. Incubation with recombinant GST-tagged Bms1^(aa1-705)^ (Figure 5A) revealed a strong and reproducible interaction between the N-terminal region of Bms1 and the WT Rcl1 protein, whereas no binding was detected for the Rcl1_RDK_ mutant.

This finding suggested that Rcl1_RDK_ might not be incorporated into the SSU processome *in vivo*. To test this, we generated a yeast strain expressing HA-tagged Rcl1 under control of the repressible *GAL10* promoter, allowing depletion of the endogenous Rcl1 protein. This strain was then transformed with plasmids encoding His_6_-TEV-protein A (HTP) tagged forms of WT Rcl1 and Rcl1_RDK_, or an empty plasmid (pRS316) and grown in minimal medium containing glucose. Rcl1-containing complexes were purified on IgG sepharose followed by TEV cleavage, and associated proteins and RNA were detected by western and northern blotting, respectively (Figure 5). This revealed that WT Rcl1 and Rcl1_RDK_ were expressed and purified to similar levels. However, association of Rcl1_RDK_ with the box C/D snoRNP-specific protein Nop1 (Figure 5B) as well as the U3 snoRNA and several pre-rRNA processing intermediates (23S, 22S/21S, 20S) (Figure 5C) was severely reduced, confirming that the Rcl1_RDK_ mutant fails to associate with the SSU processome. To support this finding, we performed immunofluorescence in cells expressing HTP-tagged WT Rcl1 and Rcl1_RDK_ (Figure 5D). WT Rcl1 localized to the nucleolus as expected, whereas Rcl1_RDK_ was mainly retained in the cytoplasm.

We conclude that pre-rRNA processing defects associated with yeast Rcl1_RDK_ are likely due to impaired nuclear import and SSU processome formation, due to the loss of binding and recruitment via Bms1.

## DISCUSSION

Here, we present data implicating Utp24 as the endonuclease responsible for early pre-rRNA cleavages at sites A1/1 and A2/2a that generate the major precursor to the 18S rRNA.

*In vivo* UV crosslinking was performed in actively growing cells to identify *bona fide* RNA targets for yeast Utp24 (Figure 1 and Supplementary Figure S2). Utp24 RNA binding was strongest in the region of the 18S rRNA central pseudoknot, a highly conserved structural unit that forms the core of the small ribosomal subunit. Additional crosslinking was observed close to site A1 and in the 5′-ETS pre-rRNA spacer region. Pseudoknot formation is guided by U3 and is required for A0, A1 and A2 cleavages (3). Strikingly, Utp24 was recovered in association with the U3 snoRNA regions that base-pair with the pre-RNA as well as with the U3-binding sites in the 5′-ETS and the 18S elements forming the proximal and distal sides of the pseudoknot (see Figure 1H). These finding indicate a role for U3 in Utp24 recruitment to the A1 cleavage site and, potentially, a role for Utp24 in verification of U3-rRNA interactions.

Utp24 was also crosslinked to the eukaryotic expansion segment 6 (ES6) within the 18S rRNA central domain (Figure 1D). The ES6 region has recently emerged as a binding hub for many ribosome biogenesis factors including the RNA helicase Dhr1, which is required to dissociate U3 from the pre-rRNA following cleavages at sites A0 and A1, and preceding A2 cleavage (35). Utp24 and Dhr1 both crosslink to the same U3 snoRNA regions, which engage in pre-rRNA interactions (35). It is possible that sequential binding of both proteins to U3 might be important to guide structural rearrangements to promote U3 release and/or positioning of Utp24 for A1 and A2 cleavages.

We did not detect clear Utp24 crosslinking around site A2 in the ITS1 sequence. We speculate that Utp24 is recruited to stable docking points within the mature 18S rRNA sequence, and only transiently interacts with the cleavage sites during catalysis. The observed coupling of cleavage at sites A1 and A2 suggests that they are brought into proximity by transient changes in pre-ribosome structure, which remain to be determined.

*In vitro* endonuclease assays revealed that recombinant Utp24 proteins from yeast and human each show activity on a pre-rRNA substrate containing yeast site A2 (Figure 2). As for other PIN domain proteins (7,25,30), this activity required Mn^2+^ ions and the conserved metal-binding amino acids of the PIN domain. Interestingly, yeast and human Utp24 not only cleaved the authentic site A2 (5′-AAC/ACAC-3′), but also at other positions in a similar sequence context (Figure 2). The cleavage pattern is identical in both proteins and therefore likely specific to the conserved Utp24 PIN domain. Very low levels of cleavage at these positions around site A2 are also observed *in vivo* (31), but they are underrepresented compared to the *bona fide* A2 site. Bound transacting factors within the SSU processome therefore likely assist the specificity of Utp24-mediated cleavage *in vivo*. The recently mapped 2a site in the human pre-rRNA (5′-C/GAC/GC-3) (19) appears to be very different to yeast A2. However, cleavage takes place at 5′C/purine3′ in both cases. We also tested RNA substrates including yeast site A1, but observed only low levels of cleavage in ACAC-rich regions within the 5′-ETS close to the U3 base-pairing sites (Supplementary Figure S3B and data not shown). Notably, the sequence context of site A1 (5′-GU/UA-3′) does not match yeast A2 or human 2a, highlighting the importance of additional factors and/or structural arrangements within the pre-rRNA that might be needed to facilitate Utp24-mediated cleavage at this site *in vivo*.

Consistent with yeast data, *in vivo* studies on human UTP24 showed that an intact UTP24 PIN domain was required for cleavage at the 5′-end of 18S (site 1) and at site 2a within the ITS1 spacer, but not at A0 or A’ in the 5′-ETS (Figure 3). The requirement for UTP24 in site 1 cleavage and the presence of an alternative pathway to process to the 5′-end of 18S in the absence of endonuclease cleavage by UTP24 was recently reported (15). However, in contrast to our results, XRN2 was not implicated in 5′-3′-exonuclease trimming to generate the aberrant 5′-end of 18S. This discrepancy might reflect the use of stable shRNA expression by Tomecki *et al*. (15), which strongly impaired cellular viability and could result in secondary effects. Notably, inspection of published northern analyses reveals phenotypes that link UTP24 to 2a cleavage (Supplementary Figure S10 in (15)).

Both Rcl1 and Utp24 have previously been proposed to cleave site A2 in yeast (4,5,9). A2 cleavage can occur either co- or post-transcriptionally, so two separate enzymes might act in these distinct contexts. The catalytic role of yeast Rcl1 in A2 cleavage was reported to be dependent on an intact RDK motif, which was proposed to mediate RNA substrate binding (9). We found, however, that the Rcl1_RDK_ mutation blocks binding to the SSU processome component Bms1 *in vitro* (Figure 5A), resulting in reduced nuclear import (Figure 5D) and the loss of stable incorporation into the processome (Figure 5B and C) *in vivo*. An intact SSU processome is required for A0-A2 cleavage potentially explaining the pre-rRNA processing defect in strains expressing Rcl1_RDK_. Mutation of the equivalent motif in human RCL1 (‘RHK’) did not affect 18S production (Figure 4). However, human SSU processome assembly appears to differ from yeast, since RCL1 associates prior to A’ cleavage, while BMS1 largely binds after cleavage at A’ (36,37).

This work has implicated Utp24 as the endonuclease responsible for two coupled pre-rRNA cleavages in 18S rRNA maturation. The enzymes responsible for cleavage at yeast and human site A0 and human A’ are probably also among the known SSU processome components, but their identities remain an intriguing mystery.

## ACCESSION NUMBERS

GEO database (http://www.ncbi.nlm.nih.gov/geo/): identifier GSE75991.

## Supplementary Material

Supplementary Data

SUPPLEMENTARY DATA
