# Supplementary material for: The PIN domain endonuclease Utp24 cleaves pre-ribosomal RNA at two coupled sites in yeast and humans
Source: Nucleic Acids Res. 2016 Mar 31;44(11):5399–409. doi: 10.1093/nar/gkw213 (PMC4914098; doi:10.1093/nar/gkw213)

SUPPLEMENTARY TABLES

Table S1: Yeast strains used in these studies

BY4741	MATa; his3Δ1; leu2Δ0; met15Δ0; ura3Δ0	(Euroscarf)
Utp24-HTP	MATa; his3Δ1; leu2Δ0; met15Δ0; ura3Δ0; UTP24-HTP-URA3	(this study)
Rcl1-HTP	MATa; his3Δ1; leu2Δ0; met15Δ0; ura3Δ0; RCL1-HTP-URA3	(this study)
Gal::rcl1	MATa; his3Δ1; leu2Δ0; met15Δ0; ura3Δ0, KAN::-pGAL1-3HA::rcl1	(this study)

Table S2: Oligonucleotides (5'-3') used in these studies

**Strain construction**	
**Utp24-HTP**	
F	GGCGGTCACGCATACGTCATTGAAAAATTGCCAGATGTCTTTGAG CACCATCACCATCACC
R	CCAAAAACATTGCACTACATACTTAAAGAGTGAATACACAGTAGTT ACGACTCACTATAGGG
**Rcl1-HTP**	
F	CAAGGGTATTGGTTTCACAAACACAAGCAAAAAGATTGCAGAGCA CCATCACCATCACC
R	ATACAGTGGTCTAATATCTATTGAATAATATTAGAACATGTACGAC TCACTATAGGG
**GAL1-3HA::rcl1**	
GAL/HA-F4	CATAAGCTACGTATGTTGTAAGGGTATAACATCTGTCAAGGAATTC GAGCTCGTTTAAAC
GAL/HA-R3	ACCCTTGGAAAGTGGTGTATTTGGGGGCGGAAGATGACATGCAC TGAGCAGCGTAATCTG
**CRAC**	
3'-Linker	5'- rAppTGGAATTCTCGGGTGCCAAGG/ddC/ -3' (miRCat-33™)
5'-Linker (L5Ab)	5'-invddT-ACACrGrArCrGrCrUrCrUrUrCrCrGrArUrCrU rNrNrNrArUrUrArGrC-OH-3'
RT	CCTTGGCACCCGAGAATT (miRCat-33™)
PCR - F	AATGATACGGCGACCACCGAGATCTACACTCTTTCCCTACACGAC GCTCTTCCGATCT
PCR - R	CAAGCAGAAGACGGCATACGAGATCGGTCTCGGCATTCCTGGCC TTGGCACCCGAGAATTCC
**Cloning**	**(Vector)**
Human	
**UTP24**	**(pcDNA5 and pGEX-6P1)**
F (Bgl ll)	CACCAGATCTATGGGGAAGCAAAAGAAAACAAGGAAGTATGCGA CCATGAAGCGAATGCTTAG
R (Xho I)	CGCGCTCGAGTTAGAATCGAGGGGCTCCATAATCATCTGG
**RCL1**	**(pcDNA5)**
F (BamH I)	CACCGGATCCATGGCGACTCAGGCGCACTC
R (Xho I)	CCCCCTCGAGTCACTTGAGGGTCTTGCTAAGGTTGG
Yeast	
**Bms1**	**(pGEX-6P1)**
F (BamH I)	CACCGGATCCATGGAGCAGTCTAATAAACAGCACCGTAAG
R (Sal I)	GTCGACTTACCTCCTCATCTTACGTGGACGAGATTC
**Rcl1**	**(pET100 and pGEX-6P1)**
F (BamH I)	CACCGGATCCATGTCATCTTCCGCCCCCAAATAC
R (Sal I)	GTCGACCTATGCAATCTTTTTGCTTGTGTTTGTG
**Rcl1-HTP**	**(pRS316)**
F (Sal I)	AGCTGTCGACCGAAACATAAAGCTGAAAGACTAG
R (Sal I)	ACGTAGTCGACGCTGGATGGGAAGCGTACC
**Utp24**	**(pGEX-6P1)**
F (BamH I)	GCGCGGATCCATGGGTAAAGCTAAGAAAACAAGAAAGTTTG
R (Sal I)	CCCCGTCGACTTAAAAGACATCTGGCAATTTTTCAATG
**Nob1**	**(pGEX-6P1)**
F (Bgl II)	CACCAGATCTATGACCGAAAACCAAACCGCAC
R (Xho I)	CTCGAGCTAACTTCTCCTTTTGGAACTGTTGACGTAC

Mutagenesis	(Mutation)
Human	
**UTP24**	**D72N**
F	CCACCTTACCACATCCTCGTTAATACCAACTTTATCAACTTTTCC
R	GGAAAAGTTGATAAAGTTGGTATTAACGAGGATGTGGTAAGGTGG
**UTP24**	**D142N**
F	CCATGTACACACAAAGGAACCTATGCAAATGACTGCTTAGTACAG AGAGTAAC
R	GTTACTCTCTGTACTAAGCAGTCATTTGCATAGGTTCCTTTGTGTG TACATGG
**RCL1**	**RHK -> AAA**
F	CGATAGAATTTTTGGCGGCTTTGGCGAGCTTTTTCCAG
R	CTGGAAAAAGCTCGCCAAAGCCGCCAAAAATTCTATCG
Yeast	
**Bms1**	**aa705 -> stop**
F	GATAATTCATTCACTAATTTTGATGCGGAGTAAAAAAAGGACTTAA CCATG
R	CATGGTTAAGTCCTTTTTTTACTCCGCATCAAAATTAGTGAATGAA TTATC
**Rcl1**	**RDK -> AAA**
F	GACGAAAGATTCATAATCCTCTTGGCAGCTATTGCGAAGATCTTTA ATACTGAAGTC
R	GACTTCAGTATTAAAGATCTTCGCAATAGCTGCCAAGAGGATTAT GAATCTTTCGTC
**Utp24**	**D68N**
F	CAATCAAGCTATAAAGCCACCTTATCAAGTACTGATAAATACCAAT TTTATAAATTTTTCTATCC
R	GGATAGAAAAATTTATAAAATTGGTATTTATCAGTACTTGATAAGG TGGCTTTATAGCTTGATTG
**Utp24**	**D138N**
F	CGCACAAGGGTACGTACGCGAATGACTGTTTAGTGCATCGAGTC
R	GACTCGATGCACTAAACAGTCATTCGCGTACGTACCCTTGTGCG
**Northern blotting**	
hITS1 (6121)	AGGGGTCTTTAAACCTCCGCGCCGGAACGCGCTAGGTAC
h18SE	CCTCGCCCTCCGGGCTCCGTTAATGATC
yU3 (snR17a)	ATGGGACTCATCAACCAAGTTGG
yD-A2	CGGTTTTAATTGTCCTA
yA2-A3	TGTTACCTCTGGGCCCCGATTG
**Primer extension**	
h1-RT	CACTGTACCGGCCGTGCG
yA1-RT	TGAGCCATTCGCAGTTTCAC
yA2-RT	GTTTGTTACCTCTGGGCCCC
yD-RT	CCATCTCTTGTCTTCTTGCCCAG
**For transcription**	
	**(yeast 35S pre-rRNA: nt2301-2844)**
y18S-200-T7-F	CGGAATTCTAATACGACTCACTATAGGGCTTGCGTTGATTACGTC CC (T7 promotor)
yITS1-R	CCAGTTACGAAAATTCTTGTTTTTGAC
	**(yeast 35S pre-rRNA: nt335-1846)**
y35S-nt335-T7F	CGGAATTCTAATACGACTCACTATAGGGAATGCCTTGTTGAATAG CC (T7 promotor)
y35S-nt1846-R	CAATTCCTTTAAGTTTCAGCCTTG

Table S3: siRNAs used in these studies

Gene	Target Sequence (5’-3’)	Source
Luciferase GL2	CGUACGCGGAAUACUUCGA	Eurofins MWG (1)
RCL1	GAACAUGACUGUAGCGUCC	Eurofins MWG
UTP24	UCCAAGAUUUGAACGAUUA	Eurofins MWG
XRN2	AAGAGUACAGAUGAUCAUG	Eurofins MWG

Table S4: Antibodies used in these studies

Target	Source	Cat. No.	Dilution	Species
**Western blotting**				
α-hKaryopherin	Santa Cruz	sc-11367	1:1000	rabbit
α-hRCL1	Eurogentec (2)	custom	1:250	rabbit
α-hUTP24	Eurogentec (2)	custom	1:1000	rabbit
α-hXRN2	Bethyl Laboratories, Inc.	A301-103A	1: 2000	rabbit
α-TAP*	Thermo Scientific	CAB1001	1:10,000	rabbit
α-yNop1	Santa Cruz	sc-57940	1:2000	mouse
α-yMtr4	Eurogentec	custom	1:10,000	rabbit
α-mouse-HRP	Santa Cruz	sc-2316	1:10,000	donkey
α-rabbit-HRP	Santa Cruz	sc-2313	1:10,000	donkey
**Immunofluorescence**				
α-TAP	Thermo Scientific	CAB1001	1:1000	rabbit
α-yNop1	Santa Cruz	sc-57940	1:2000	mouse
α-rabbit-IgG (Alexa Fluor 555 conjugate)	Invitrogen	A-31572	1:500	donkey
α-mouse-IgG (Alexa Fluor 555 conjugate)	Invitrogen	A-31570	1:500	donkey

***** The α-TAP antibody recognizes the C-terminus of the HTP construct before and after TEV cleavage.

SUPPLEMENTARY FIGURE LEGENDS

Figure S1: Ribosome biogenesis pathways in yeast and humans

A, B Key steps in the pre-ribosomal RNA processing pathways in *S. cerevisiae* **(A)** and *H. sapiens* **(B)**. Cleavages important for 18S rRNA processing are indicated. RNA intermediates of the minor, 2a-dependent human pathway that are accumulating in the absence of XRN2 (30SL5', 36S and the ITS2 fragment) are highlighted in red. The positions of radiolabeled probes used for primer extension (yA1-RT, yD-RT, yA2-RT and h1-RT) or northern blotting (yD-A2, yA2-A3, h18SE and hITS1) are marked above the primary transcripts. ETS: external transcribed spacer; ITS: internal transcribed spacer.

Figure S2: *In vivo* RNA-protein crosslinking studies (CRAC) to define binding sites for Utp24 and Rcl1

A Outline of the CRAC crosslinking technique.

B Proteins purified from UV-crosslinked yeast strains expressing either C-terminally HTP-tagged Utp24 or Rcl1 were separated by SDS-PAGE and visualized by immunoblotting using the anti-TAP antibody (left panel), which recognizes the C-terminus of the HTP construct after TEV cleavage. Radioactively labeled crosslinked RNA fragments were detected by autoradiography (right panel).

C Mutations and microdeletions on the 5'-end of the 18S rRNA (nucleotides 1-20), representing precise Utp24 binding sites from two replicate experiments, are plotted as deletions per million mapped sequences. The position of the mature 18S rRNA and site A1 are indicated on the left.

Figure S3: Recombinant yeast Utp24 and Nob1 exhibit specific *in vitro* cleavage activities in the presence of 5 mM Mn^{2+} at sites A2 and D, respectively.

A Recombinant, GST-tagged wild type or mutant yeast Utp24, wild type yeast Rcl1 or wild type and mutant human UTP24 proteins were expressed in *E. coli* and purified on a glutathione sepharose column in the presence of 1 mM Mn^{2+}. The N-terminal GST tag was removed by prescission protease (PP) cleavage and the proteins separated by SDS-PAGE and stained with Coomassie blue. Asterisk: free GST-tag.

B *In vitro* transcribed RNA mimicking a 5'-ETS-18S pre-rRNA fragment before A0 and A1 cleavage (yeast 35S pre-rRNA: nt335-1846) was incubated without recombinant protein, wild type or mutant yeast Utp24 protein in the presence of 5 mM Mn^{2+} and analyzed by primer extension. The position of the primer (yA1-RT) is shown. Non-treated RNA substrate was used to generate a sequencing ladder. Recombinant Utp24-mediated cleavages in the 5'-ETS are marked by asterisks.

C Recombinant, GST-tagged wild type yeast Nob1 was expressed in *E. coli*, purified on a glutathione sepharose column in the absence of Mn^{2+} and analyzed by SDS-PAGE and Coomassie staining. Uncleaved, GST-tagged yNob1 protein was used in the cleavage assays shown in panel **D**.

D *In vitro* transcribed RNA mimicking a pre-rRNA fragment before A2 and D site cleavage (yeast 35S pre-rRNA: nt2301-2844) was incubated without recombinant protein, wild type or mutant yeast Utp24 or wild-type Nob1 protein in the presence of 5 mM Mn^{2+} and analyzed by primer extension. The positions of the primers yA2-RT (left) and yD-RT (right) are shown. Non-treated RNA substrate was used to generate a sequencing ladder. Recombinant protein-mediated cleavages at and around sites A2 and D are marked by arrows and asterisks, respectively.

Figure S4: Wild type and mutant recombinant proteins exhibit *in vitro* cleavage activity at site A2 in the presence of 10 mM Mg^{2+}

A Recombinant wild type or mutant yeast Utp24 and wild type yeast Rcl1 proteins as in **Figure S3A** were dialyzed into buffer MKG (50 mM MOPS/KOH pH 7.5, 200 mM KCl, 10 % glycerol, 1 mM DTT, 1 mM THP), and analyzed by SDS-PAGE and Coomassie staining.

B Equal amounts of wild type or mutant yeast Utp24 or wild-type Rcl1 proteins in buffer MKG (see panel **A**), or no protein, were incubated for 1h at 30°C with *in vitro* transcribed RNA mimicking a pre-rRNA fragment before A2 cleavage (yeast 35S pre-rRNA: nt2301-2844). Here, reactions were carried out in the presence of 10 mM Mg^{2+} in 50 mM MOPS/KOH pH 7.5, 100 mM KCl, 2 mM dithiothreitol, 100 μg ml^{-1} bovine serum albumin, 0.8 unit μl^{-1} RNasin, 5% glycerol and 10 ng μl^{-1} *E.coli* tRNA. 10 μl reactions containing ~20 pmol of protein were pre-incubated for 20 min at 30°C before adding 0.125 pmole of the RNA substrate, which was then analyzed by primer extension using primer yA2-RT. Recombinant protein-mediated cleavages at site A2 are marked by an arrow.

Figure S5: Protein and RNA analysis of UTP24 RNAi-rescue cell lines

A HEK293 cells as in **Figure 3** were stably transfected with plasmids expressing the FLAG tag alone (pcDNA5) or wild type (WT) or mutant forms of FLAG-UTP24 (D72N, D72N/D142N). The FLAG-UTP24 cDNA sequence had been modified to render the mRNA resistant to the UTP24 siRNA. Protein extracted from control cells (GL2), or those depleted of endogenous UTP24 (UTP24), XRN2 (XRN2) or both (X+24) by RNAi, was separated by SDS-PAGE and transferred to nitrocellulose membrane. Protein levels were analyzed by immunoblotting using antibodies specific for UTP24 and XRN2, or Karyopherin as loading control. The asterisk denotes a non-specific protein recognized by the antibody.

B RNA levels from **Figure 3B** were normalized to the 47S/45S pre-rRNAs and plotted for each GL2 (grey) or UTP24 (black) knockdown. The identity of each peak is indicated.

C RNA extracted from HEK293 cell as in panel **A** was analyzed by primer extension using a probe to detect the 5'-end of 18S (h1-RT). Total RNA from cells expressing the FLAG tag alone (pcDNA5), treated with the control siRNA GL2, was used to generate a sequencing ladder. Positions of the natural site 1 and 2 nt downstream are indicated on the left.

Figure S6: Protein analysis of RCL1 RNAi-rescue cell lines

A HEK293 cells as in **Figure 4** were stably transfected with plasmids expressing the FLAG tag alone (pcDNA5) or wild type FLAG-RCL1 (WT) or the FLAG-RCL1-RHK mutant (RHK). The FLAG-RCL1 mRNAs were rendered resistant to the RCL1 siRNA. Protein extracted from control cells (GL2), or those depleted of endogenous RCL1 (RCL1), XRN2 (XRN2) or both (X+R) by RNAi were analyzed by immunoblotting using antibodies specific for RCL1 and XRN2, or Karyopherin as loading control. The asterisk denotes a non-specific protein recognized by the antibody.

B RNA levels from **Figure 4A** were normalized to the 47S/45S pre-rRNAs and plotted for each GL2 (grey) or RCL1 (black) knockdown. The identity of each peak is indicated.

SUPPLEMENTARY REFERENCES

1. Elbashir, S.M., Harborth, J., Weber, K. and Tuschl, T. (2002) Analysis of gene function in somatic mammalian cells using small interfering RNAs. *Methods*, **26**, 199-213.
2. Sloan, K.E., Mattijssen, S., Lebaron, S., Tollervey, D., Pruijn, G.J. and Watkins, N.J. (2013) Both endonucleolytic and exonucleolytic cleavage mediate ITS1 removal during human ribosomal RNA processing. *J Cell Biol*, **200**, 577-588.

Wells *et al.*, Figure S1

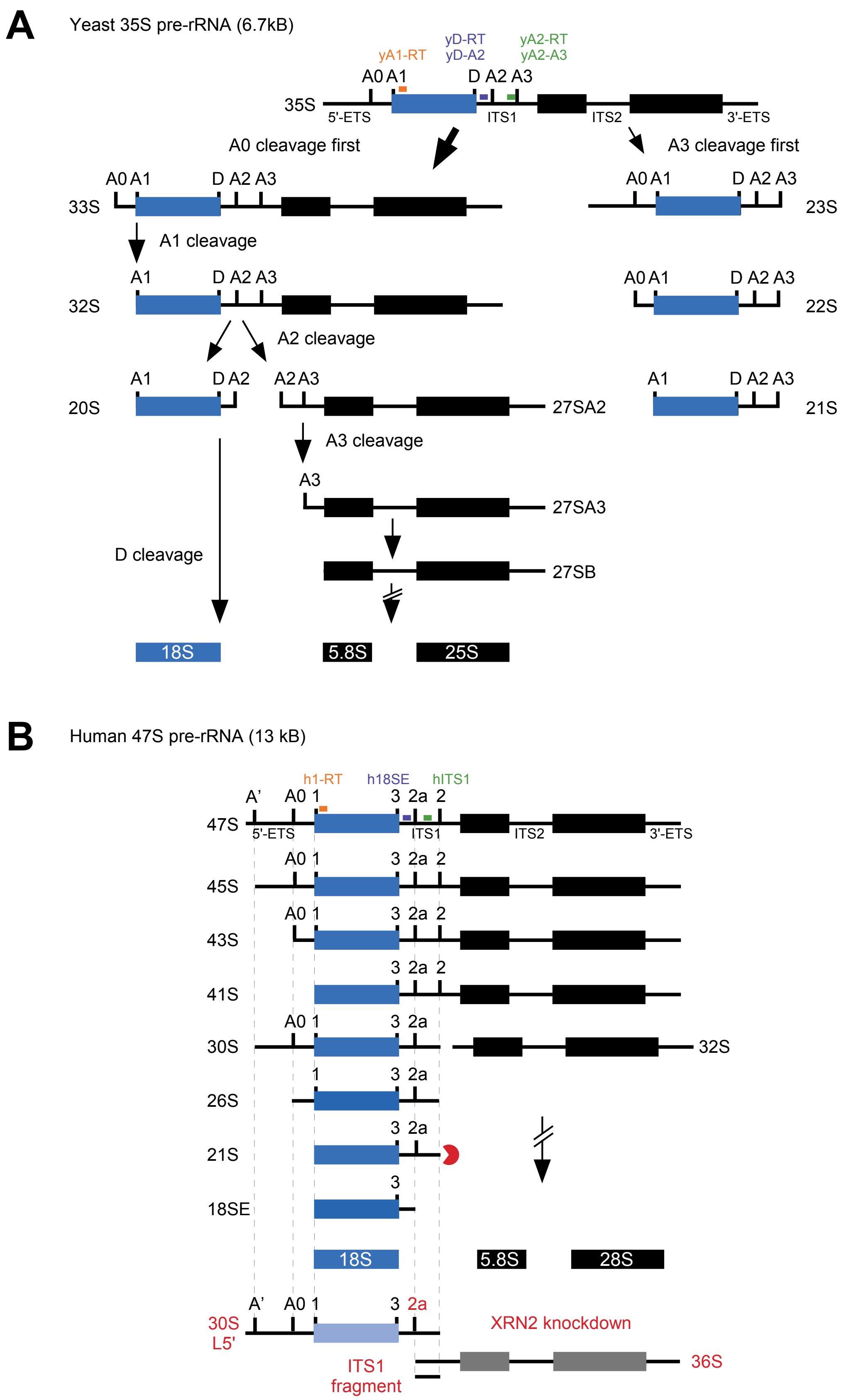

Wells *et al.*, Figure S2

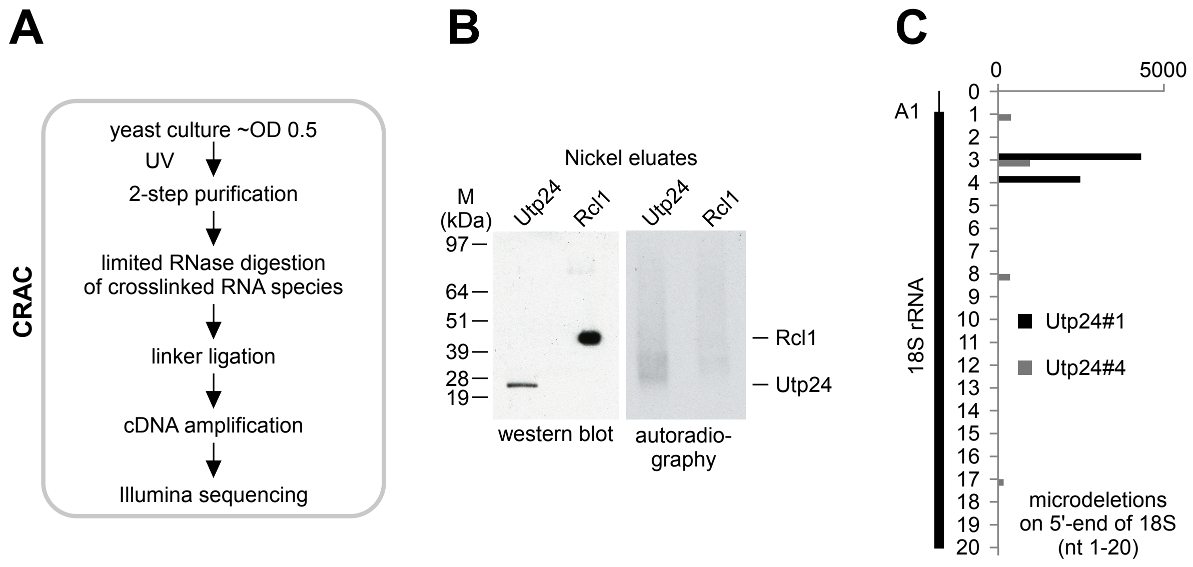

Wells *et al.*, Figure S3

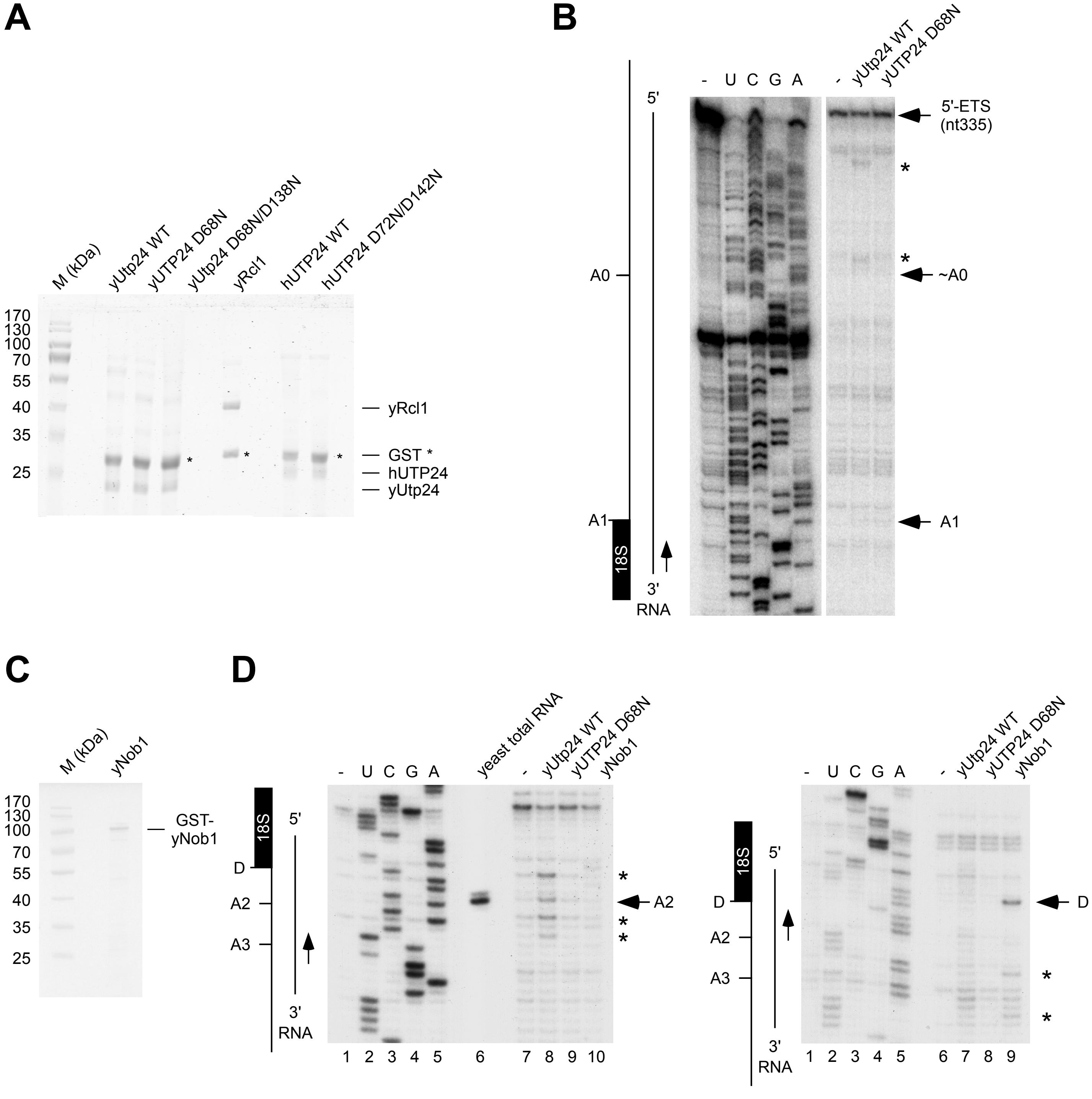

Wells *et al.*, Figure S4

A

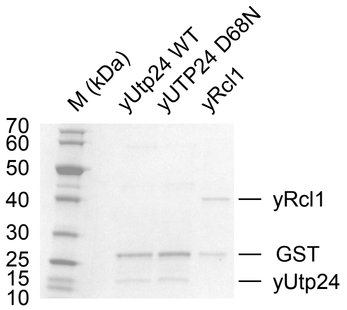

B

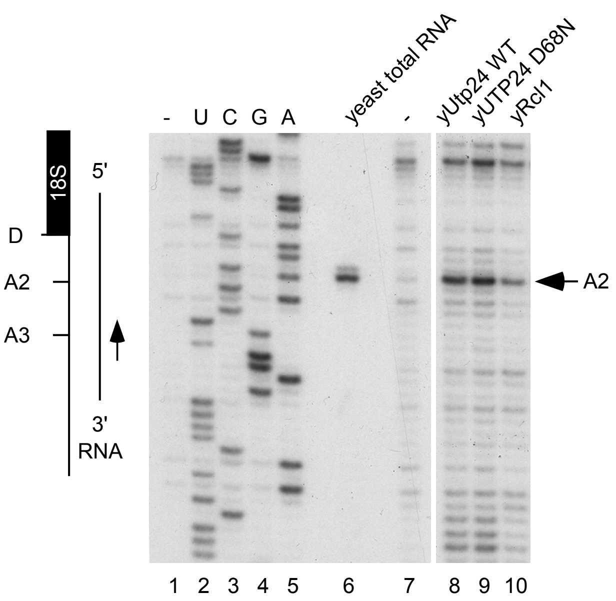

Wells *et al.*, Figure S5

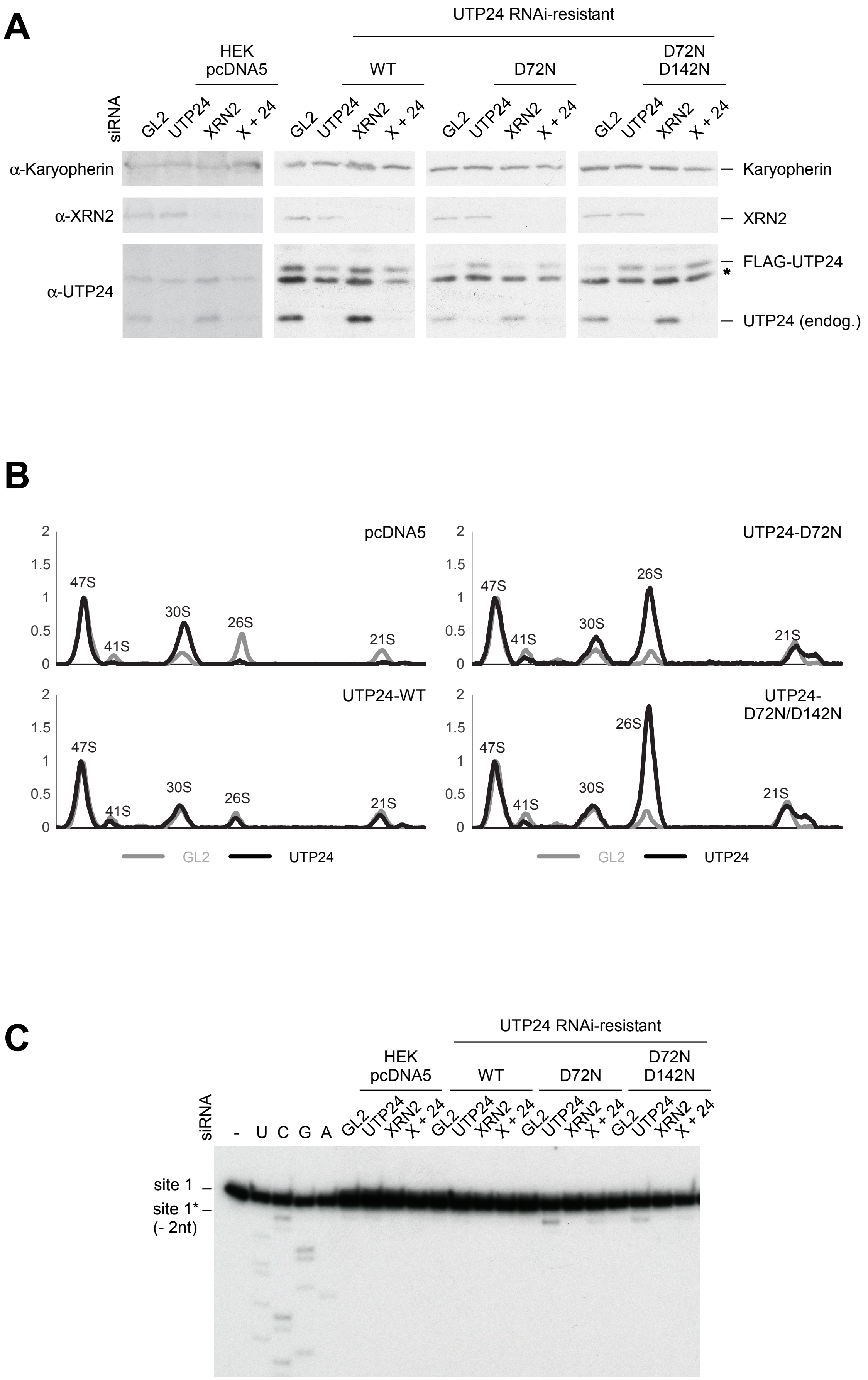

Wells *et al.*, Figure S6

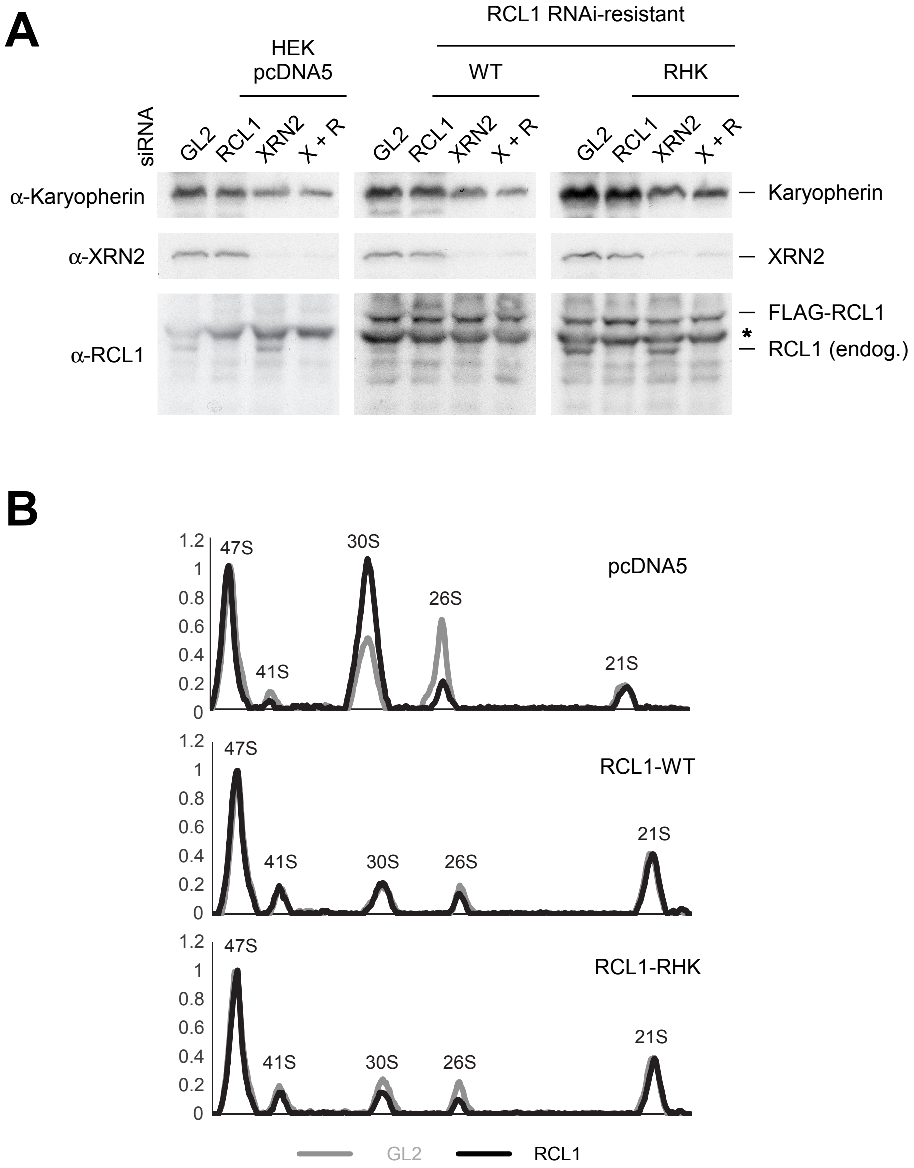

Supplement: Supplementary Data [file gkw213_Supplementary_Data.zip › nar-00061-r-2016-File003.pdf]
