# Supplementary material for: The PIN domain endonuclease Utp24 cleaves pre-ribosomal RNA at two coupled sites in yeast and humans
Source: Nucleic Acids Res. 2016 Mar 31;44(11):5399–409. doi: 10.1093/nar/gkw213 (PMC4914098; doi:10.1093/nar/gkw213)
Supplement: SUPPLEMENTARY DATA [file supp_44_11_5399__index.html]

The PIN domain endonuclease Utp24 cleaves pre-ribosomal RNA at two coupled sites in yeast and humans — The PIN domain endonuclease Utp24 cleaves pre-ribosomal RNA at two coupled sites in yeast and humans — SUPPLEMENTARY DATA 

# The PIN domain endonuclease Utp24 cleaves pre-ribosomal RNA at two coupled sites in yeast and humans

## SUPPLEMENTARY DATA

- SUPPLEMENTARY DATA
